# Thank you to our 2024 reviewers

**DOI:** 10.1016/j.eclinm.2025.103134

**Published:** 2025-02-24

Peter Aaby

Ali Abbasian Ardakani

Hazreen Abdul Abdul Majid

Rizwan Abdulkader

Hairil Abdullah

Jithma Abeykoon

L G Abreu

Usman Abubakar

Mohammad H Abukhalil

Laith Abu-Raddad

Andres Acosta

Lisa Adams

Antoine Adenis

Muwonge Adrian

Niloofar Afari

Kingsley Agho

A J Agopian

Kedir Yimam Ahmed

Saifuddin Ahmed

Yasar Ahmed

Jaeil Ahn

Katherine A Ahrens

Noor Ul Ain

Jasmini Alagaratnam

Ziyad Al-Aly

Uazman Alam

Sarah Alexander

Joachim Alexandre

Fernando Alfonso

Diallo Alhassane

Jaume Alijotas-Reig

Jessica Allegretti

Hesham Al-Mekhlafi

Cristina Alonso-Vega

Mohammad S Alrashdan

Moath Alsaqaaby

Claudia Alves

Ian Patrick Joseph Alwayn

Frédéric Amant

Sergio Amaro

Faizan Amin

Yuichi Ando

Tiing Leong Ang

Emanuele Angelucci

Andrea Angheben

Takatoshi Aoki

Deepa Arachchillage

Stella Arakelyan

Anna Lisa Arcari

Livia Archibugi

Alvaro Arjona-Sánchez

Urska Arnautovska

Mekinian Arsene

Rob J W Arts

Samaneh Asgari

Abolfazl Avan

Abraham Avigdor

Baffour Awuah

Paul T Y Ayuk

Andrew Azman

Marianne Aznar

Tomohisa Baba

Vanessa Babineau

Thomas Bachelot

Andrea Bacigalupo

Martina Badell

Wolfgang Baeumler

Luis Bahamondes

Anees Bahji

Shasha Bai

Stephen Bain

Tomas Baka

Haval Balata

Lia Bally

Hideaki Bando

Aduragbemi Banke-Thomas

Hossein Bannazadeh Baghi

Yanping Bao

Stephane Bar

Biagio Barone

Savio Barreto

Aluisio Barros

Karen Bartholomew

Alfred Bartolucci

Rosaleen Baruah

Enrico Baruffini

Renato Bassan

Daniela Basso

Sriparna Basu

Kaitlin Batley

Malcolm Battin

Lisa Baumann Kreuziger

Yvonne Baumer

Iacopo Baussano

Francisco Jose Bautista

Abdul-Hamid Bazarbachi

Eliza Beal

Richard Beasley

Anna Becker

Jacqueline Becker

Laurel A Beckett

David Belada

Sabine Belard

Smaranda Belciug

Jennifer Bell

Riccardo Bellazzi

Jade Benjamin-Chung

Julie Bennett

Joana Berger-Estilita

Veerle Bergink

Christina Bergmann

Johannes Berkhof

Johannes Berkhof

Carola Berking

Inge Bernstein

Jane Bertrand

Christopher Beynon

Julien Bezin

Nita Bhandari

Dharma Bhatta

Kam Bhui

Nan Bi

Giuseppe Bifulco

Marie-Noëlle Billard

Alexandra Blackwell

Karl Blanchet

Jean-Yves Blay

Katherine Bline

Paul J N Bloem

Adrian Bloor

Cauane Blumenberg

John Edward Blundell

Sandeep Bodduluri

Martin Boeree

David Boettiger

Giorgio Bogani

Saskia Bogers

Isaac Bogoch

Michael Böhm

Anders Boisen

Maryline Bonnet

Juan Bornman

Josep Maria Borras

Pau Bosch-Nicolau

Matthias Bossard

Arnaud Bourdin

Antoine Boutet

Andrea Bradford

Daan Brandenbarg

Ola Bratt

Paula Braveman

George Bray

Michael Joel Brenner

John Bridges

Anne Craveiro Brøchner

F J M Broekmans

Jacques Brown

Timothy Brown

Nele Brusselaers

Nele Brusselaers

W Chris Buck

Pip Buckingham

Diana Buist

Andrea Buono

Danilo Buonsenso

John Burn

Tom Burne

William Busse

Warwick Butt

Anthony Byrne

Susan C Byrne

Susan Calcaterra

Peter M Calhoun

Daniel Candotti

Suzanne Cannegieter

Dexter Canoy

Guiying Cao

Xin-xin Cao

Giuseppina Capra

Evie H Carchman

Robert G Carlson

Suzan Carmichael

Thomas J Carmody

Pricivel Carrera

Sylvine Carrondo Cottin

Ben Carter

Rosa Casas

Peter Cashin

Mariana Concepcion Castells

Matthew Cavanaugh

Nurdan Çay

Arthur Caye

Dario Cazzoli

Emanuele Cencini

Antonio Cerasa

Anna Ceraso

Magdalena Cerda

Paige E Cervantes

Krishnaraj Chadaga

Victoria Lee Champion

Joe Kwun Nam Chan

Ngan Yin Chan

Polin Chan

Tu-Hsuan Chang

Wing Chung Chang

Charlotte Charpentier

Chan Yoon Cheah

Helen Chen

Hong-Lin Chen

Huilong Chen

Hung Chou Chen

Jeng-Wen Chen

Jie Chen

Jing Chen

Jun Chen

Kai Chen

Minjiang Chen

Mu-Hong Chen

Sijing Chen

Tianmu Chen

Xiaosong Chen

Yiming Chen

Yunbo Chen

Li Cheung

Moira Shang Mei Cheung

Wisit Cheungpasitporn

Lydia Chevalier

Helen Cheyne

Rajkumar Chinnadurai

Hung Yi Chiou

Mohammod Jobayer Chisti

S F Cho

Horace Choi

Kangho Choi

Serah Choi

Won-Mook Choi

Elaine Chow

Mikkel Christensen

Wan–Long Chuang

Annalisa Ciabattini

Michele Ciccarelli

Irfan Cicin

Francesco Cinetto

Emilia Cirillo

Nicola Cirillo

Ralf Clemens

Tammy Clifford

Ashley Kieran Clift

Pierluigi Cocco

Paul Cockwell

Jennifer Cohen

Romain Cohen

Tim Colbourn

Intira Collins

Raffaella Colombatti

Juliet Compston

Andrew Copas

Alberto Cordero

Germaine Cornelissen

Rosie Cornish

Alessio Correani

Vera Costa

James David Cotter

Elizabeth Coulthard

Allan Covens

Aimee Crago

Tamaryn Crankshaw

Sue Crengle

Clifford L Cua

Chuangliang Cui

Antonio Cuneo

Giuseppe Curigliano

Melissa Cyperski

Lucette Cysique

Leticia Czepielewski

Omid Dadras

Xiaochen Dai

Yunfeng Dai

Garima Dalal

Stephane Dalle

Lia D'Ambrosio

Nick Daneman

Jun Dang

Sandra D'Angelo

Ziyaad Dangor

Maria Giovanna Danieli

Paola Daniore

Sarah Danson

Alain Dardashti

Patrice Darmon

Pietro Dattolo

Mellar P Davis

Jeanette Dawa

Eran Dayan

Antonio de Arriba-Muñoz

Mark A de Belder

Francesca De Felice

Ugo De Giorgi

Sara de la Salle

Daniele De Luca

Nicolò de Pretis

Andrea De Vito

Lucia Del Mastro

Alejandro D De-La-Sierra

José Delgado Alves

Maurizio Delvecchio

Jenna Demedis

Quentin Denost

Devendra Desai

Julia Dettinger

Shyamali Dharmage

Keertan Dheda

Guido Di Dalmazi

Violante Di Donato

Daniele Di Lernia

Vito Di Marco

Karidia Diallo

Michael Dibley

Alassane Dicko

Joakim Dillner

Andrea Di-Marco

Tao Ding

Joachim Dissemond

Matthew Dodd

Jennifer Doherty

Pere Domingo

Qian Dong

Yanhui Dong

Daniel A Donoho

Michelle Doose

Gregory Dore

Maria R D'Orsogna

Johannes Dorst

Lars Ove Dragsted

Alex Dregan

Dongping Du

Lingbin Du

Yuncheng Du

Guillaume Ducarme

Matthew Durstenfeld

Jean Claude Dusingize

Laura Dwyer-Lindgren

Helena Earl

Charles Eaton

Linda Oneal Eckert

Panagiota Economopoulou

Julien Edeline

Andrew Edmonds

Robert Jeffrey Edwards

Jan Egger

Laya Ekhlaspour

Nady El Hajj

Ahmed Elazab

Mohamed El-Kassas

Ayse Ercumen

Cornelia Erfurt-Berge

Masahiro Eriguchi

Gerard Espinosa

Vicente Estrada

Paolo Eusebi

D Gareth Evans

Ido Didi Fabian

Stephen Fadem

Gian Paolo Fadini

Delisa Fairweather

Adegoke Falade

Titilola Falasinnu

Maria Falkenberg

Mengjie Fang

Shencun Fang

Eric Faragher

Michael Farrell

Forough Farrokhyar

Alfonso Fasano

Martin Fassnacht

Laurent Fauchier

Seena Fazel

Artur Fedorowski

Michael Fehlings

Eleonora Feletto

Yadong Feng

J Fernadnez

Carlos Fernandez-Granda

Giovanna Ferraioli

Marc Ferrante

Clarissa Ferrari

Carlos Gil Ferreira

Luca Ferretti

John Field

Guido Filler

Neil N Finer

Günther Fink

Luca Fiorillo

Kevin Fiscella

Jane Fisher

Robert Fitridge

Felicity Fitzgerald

Oliver Fitzgerald

Jennifer Flemming

Lena Flyckt

Joseph Flynn

Daniele Focosi

H Rui Martins Fonseca

Nathan P Ford

Marco Fornaro

Theodoros Foukakis

David Fraile-Navarro

Joel Francis

Svein Otto Fredwall

Mason Freeman

Toby Freeman

Daniel Friedman

Carsten Friedrich

Christoph M Friedrich

Giovanni B Frisoni

Nikolaj Frost

Petter Frühling

Pin-kuei Fu

Sirui Fu

Ahmad Fuady

Naoto Fujiwara

Ramsay Fuleihan

Nancy Fullman

Tamas Fulop

Jennifer Furin

Ognjen Gajic

Mary Galea

Eva Galiza

Brenda Gallie

Marialuisa Gandolfi

Ron Gansevoort

Xin Gao

Xing-Hua Gao

Juan García-Iglesias

Luis Garcia-Marcos

Dagmar García-Rivera

Karim Gariani

Leanne Gauld

Kelly Gebo

Marco Geraci

Paul Gerdhem

Jurgen Germann

Morie A Gertz

Tamer Gheita

Uday Ghoshal

Evangelos Giamarellos-Bourboulis

Malick Gibani

Nicolae Gică

Johannes Giesinger

Clare Gilbert

Luis Andrés Gimeno-Feliu

Mika Gissler

Marco Giussani

Michael J Glantz

Stanton Glantz

Nancy Glass

Andreas Gleiss

Bengt Glimelius

Lise Gluud

Michael Gnant

Don L Goldenberg

Jason Goldman

Delia Goletti

Marc Gollub

Chris Gomez

Francesc Xavier Gomez-Olive Casas

Elise Gomez-Sanchez

Anthony Gonçalves

Tomas Jose Gonzalez-Lopez

Annekathryn Goodman

Laura Goodwin

Felicity Goodyear-Smith

Jiangtao Gou

Guillaume Goudot

Alpesh Goyal

Graeme A Graeme Currie

Norbert Graf

David Graham

Sonia Grandi

Alessandro Granito

Nick Graves

Gwenaelle Gravis

Kirsty Gray

Gian Luca Grazi

Lorraine Greaves

Joseph Greer

Jean-Christophe Gris

Erica Grodin

Henk Groen

Alessandro Gronchi

Beatriz Gros

Charles Grose

Andrew Grulich

Gerhard Gründer

Song Gu

Joshua Guedalia

Joanna Guimarães

Roman Gulati

Martin Gulliford

Jan Gunst

John Gunstad

Lingyun Guo

Pi Guo

Vivian Yawei Guo

Yi Guo

Arjun Gupta

Trish Hafford-Letchfield

Judith Hahn

Michael Hall

Wayne Hall

Rami Hallac

David Halpin

Robert Hamilton

Trevor D Hamilton

William Hamilton

Anne Hammer

Liyuan Han

Seung Hyeok Han

Tomáš Hanke

Isabel Hanson

Mohamed Hany

Elaine Hargreaves

Michael Harhay

Elinor Harriss

Caroline Hartley

Kiyoshi Hasegawa

Cesare Hassan

Peter Hasselblatt

Joanne Haviland

Jane M Hawdon

Michael Hawkes

Maria Hawkins

Harrison Hawley

Junichiro Hayano

Robert J Hayashi

Frances Hayes

Mert İlker Hayıroğlu

Jing He

Xuefeng He

Yulei He

Kurt Hecher

Tanja Hechler

Darren Hedley

Eetu Heervä

Bettina Heidecker

Oskari Heikinheimo

Allison J Hempenstall

Stevie Hendriks

José P S Henriques

Ju-sun Heo

Ramon Hermida

Jon Heron

Brock Hewitt

Takashi Higuchi

Andrew Hill

Kenji Hirata

Yusuke Hiratsuka

Fred R Hirsch

Martin Hoenigl

Jens Hoeppner

Caroline Homer

Thomas Hone

Chien Tai Hong

Jiang Hong

Namki Hong

Elske Hoornenborg

Ashley Hopkins

Hidehito Horinouchi

Biljana N Horn

Benjamin Horne

Apollinaire Horo

Apollinaire Horo

Ali Hosseininasab

Xiaotong Hou

Louise Howard

Martin R. Howell

Chinghua Hsieh

Chih-Wei Hsu

Shuhsien Hsu

Chang Hu

Xiaofei Hu

Xin Hu

Zhibin Hu

Bingsheng Huang

Chuin Chia Huang

Hairong Huang

Kewu Huang

Liang Huang

Qiong Huang

Yafang Huang

Yi-Hsiang Huang

M Mamun Huda

Robert Hüneburg

Ivan F N Hung

Gillian Hunt

Katherine Hunzinger

Christopher Hurst

Susanna Hutajulu

Julie Huynh

Woo Jin Hyung

Andrea Iaboni

Raheel Iftikhar

Peter Igaz

Sho Iketani

Irena M Ilic

Marta Imamura

Lawrence Impey

Leeberk Raja Inbaraj

Caleb H Ing

Norimitsu Inoue

Ana-Maria Iosif

Stephanie Irving

Adam Isaac

Petros Isaakidis

Arda Isik

Md Mazharul Islam

Md Monirul Islam

Md Rabiul Islam

Md Saiful Islam

Sheikh Mohammed Shariful Islam

Khalida Ismail

Erwin Ista

Per Iversen

Yoshitaka Iwanaga

Swaminathan Iyer

Shigeko Izumi

Koji Izutsu

Caroline Jackson

David A Jackson

Thomas Jackson

Bart Jacobs

Caron Jacobson

Shabbar Jaffar

Mathieu Jagt

Mamta K Jain

Marianne Uhre Jakobsen

Rajiv Jalan

Chinmay Jani

Kajsa Järvholm

Channa Jayasena

Johan Jendle

Jeffrey Jensen

Han-gil Jeong

José A Jerónimo

Nicholas Jewell

Rachel Jewkes

Hanyu Jiang

Yu Jiang

Fengyi Jin

Yong-yeon Jo

Eva Dora João

Christoffer Johansen

Asif M Johar

Douglas Johnson

Kara Johnson

Yvonne Joko-Fru

Christopher Jones

Daryl Jones

Erika Jones

Vita Jongen

Rudolf Jörres

Easter Joury

Shenghong Ju

Steven Julious

Andrew Jull

Juhani Junttila

Carla Justiniano

Zubair Kabir

Hiroshi Kagamu

Yoshihiro Kakeji

Georgios Kalambokis

Swathi Kaliki

Moses Kamita

A B M Kamrul-Hasan

Jia-Horng Kao

Ellen Kapiteijn

Samuel Kariuki

Lina Karout

Jaro I Karppinen

Ozgur Kasapcopur

Gertjan Kaspers

Masako Yano Kataoka

Ken Kato

Yasuyuki Kato

Antonis Kattamis

Howard M Katzenstein

Yoshikuni Kawaguchi

Mustafa Kayyali

Ashish Kc

Lu Ke

Karen Keddy

Dorothy Keefe

Sylvia Kehlenbrink

Jennifer Keiser

Peter Kelleher

Helen Kelly

Michael Kelly

Mark Kelson

Zoe Kelson

Cormac Kennedy

Eilis Kennedy

Kevin Kensler

Alexander Ross Kerr

Stephen Kerr

Maroof Khan

Md Nuruzzman Khan

Safi Khan

Farhana Khanam

Sahil Khanna

Ali Khashan

Preben Kidmose

Kelley Kidwell

Carlotte Kiekens

Han-Joon Kim

Jung-Sun Kim

Pirkka Kirjavainen

Taro Kishi

John Kisiel

Masafumi Kitakaze

Hiroshi Kitoh

Coleen Kivlahan

Petra Kleiblova

Eric Klein

T Klockgether

Emily R Ko

Takahide Kodama

Fumitaka Koga

Brandon A Kohrt

Masahiro Kohzuki

Loick Pradel Kojom Foko

Christine Kolbe-Stuart

Guilan Kong

Evangelos Kontopantelis

Ilan Jasper Nader Koppen

S N Kotlyarov

Ewout Kouwenhoven

Tobias Kowatsch

Henry Kranzler

Marcin Krawczyk

Marek Kretowski

Sandro Krieg

Helle Kristensen

Miltiadis Krokidis

Ilari Kuitunen

Anand Kulkarni

Nitya Kumar

Goorak Kwon

Ilias Kyriopoulos

Ville Kyto

Ioannis Kyvernitakis

Chris Labaki

James Lachaud

Curtis Lachowiez

Luke Joseph Laffin

Desiree LaGrappe

Adi Lahat

Chih-Cheng Lai

Grégory Lailler

Mitja Lainscak

Sham Lal

Grace Lam

Vincent Wai To Lam

Shannon Lange

Nina Langeland

Kamran Lankarani

Stephen Lapinsky

James Larkin

Simona Lattanzi

Eric Lau

Lucas Lauder

Jeffrey Laurence

Jose Laurent

Paolo Lauriola

Catharina Lavebratt

Kimseng Law

Laurence Le Cleach

Justin Leach

Michael Lean

Charles Leath III

Zohar Lederman

Bong-Ki Lee

Jeannette Lee

Ji-Hyang Lee

Maria Lee

Maw Sheng Lee

Pyng Lee

Tara Lee

Tsung-Hsien Lee

Jean Christophe Lega

Cristina Legido-Quigley

Véronique Le-Guern

Vicky Lehmann

Daniel Leightley

Anja Leist

Marc Leone

Angela Leung

Marcel Levi

Stephen Levine

Antonin Levy

Russell Edward Lewis

Da Li

Fu-Rong Li

Hecheng Li

Jingxin Li

Lixin Li

Molly Li

Shuya Li

Shuyan Li

Yongze Li

Yongze Li

Zixiao Li

Ioannis Liampas

Fei Liang

Lucas Liaudet

Winston S. Liauw

Anna Marina Liberati

Ka Keat Lim

Sun Min Lim

Yen Ying Lim

Chen-Yuan Lin

Yuan Lin

Martin Lindgren

Raghu Lingam

Annemiek J Linn

Mark Litzow

Fei Liu

Hanli Liu

Jiaye Liu

Jue Liu

Jufen Liu

Junting Liu

Minhui Liu

Si-Yang Liu

Tao Liu

Weiping Liu

Xiaoli Liu

Xiaoqin Liu

Yong Liu

Philippe André Liverneaux

Per Ljungman

Serigne Lo

Andrew Loblaw

Robin Lockridge

Samantha Loi

Anna Lokshin

Fortunato Lombardo

Carlos López-Gómez

Giuseppe Losurdo

Yair Lotan

Mohamed Lounis

Santiago Lozano-Calderon

Lin Lu

Shun Lu

Wenwei Lu

Stefano Luminari

Sebastian Lunke

Xiangde Luo

Angela Lupattelli

Markus Luster

Janet Lydecker

Mark Lythgoe

Bingxin Ma

Xuelei Ma

Ya'nan Ma

Yuan Ma

Michiel Maas

Manuel Macía

Graeme MacLennan

Peter MacPherson

Peter MacPherson

Clare MacRae

Finlay Alistair Macrae

Sten Madsbad

Shin Maeda

Claudio Maffeis

Elisa M Maffioli

Aurelio Maggio

Mathieu Maheu-Giroux

Benjamin Maier

Hisaki Makimoto

Gathsaurie Malavige

Amyn Malik

Luc Mallet

Laura Malone

Monica Malta

Lucia Mangone

Laurens Manning

Anand Manoharan

Mohammad Ali Mansournia

Anthony Muchai Manyara

Tsegahun Manyazewal

Ning Mao

Wenjian Mao

Ying Mao

Giulia Marchetti

Olivier Marcy

David Joel Margolis

Daniela Mariosa

Guy Marks

Michael Marks

Norbert W Marschner

Erika Martinelli

Marianne Martinello

Javier Martinez-Trufero

Giovanni Martinotti

Manoela Martins

Ana Marusic

Bruno Masquelier

Greta Massetti

Maura Massimino

Nobuaki Matsubara

Koji Matsuo

Christiane Matuschek

Joan Maurel

Oscar Mayer

Gherardo Mazziotti

Colin McArthur

Valerie McCormack

Peter McCulloch

Robert McGovern

Zachary J McKenna

Sally McManus

Anna L McNaughton

Stuart James Mealing

Benjamin Meder

James E Meiring

Tomer Meirson

Erik Melén

Giuseppinella Melita

Gabriel E Mena

Nahum Mendez-Sanchez

Nan Meng

Ruogu Meng

Xiangming Meng

Xiaochun Meng

Zhu Meng

Fiona Mensah

Nicolas Menzies

Jon Merz

Maximilian Merz

Carel G M Meskers

Sarah Messiah

Janey Messina

Jeffrey Meyer

Thomas J Meyer

Paul Meyers

Badar Mian

Thomas Michaeli

Timothy I Michaels

Federica Miglietta

Filippo Migliorini

Robert J H Miller

Edward Mills

Geltrude Mingrone

Lidia Mínguez-Alarcón

Shona Minson

Alexander Dimitri Miras

Kamilla Woznica Miskowiak

Hannah Mitchell

Mostafa Hossam El Din Moawad

Sebastian Moguilner

Noushin Mohammadifard

Sneha Mohan

Babak Mohit

Ghulam Mohyuddin

Kevin Monahan

Giovanni Monteleone

Francesco Montorsi

Luis M Montuenga

Alan Moody

Diego Moretti

Abraham Morgentaler

Mario Luca Morieri

Keita Morikane

Takeshi Morimoto

Dion Morton

Yakui Mou

Sizulu Moyo

Elias Mpofu

Mwenya Mubanga

Maximilian Muenchhoff

Somnath Mukherjee

Rudzani Muloiwa

Georgina Mulundu

Ghina Mumtaz

Isabelle Munyangaju

Michio Murakami

Edoardo Muratore

Srinivas Murthy

Daniel Musher

Godfrey Musuka

Victoria Mutambara

David Mutch

Sharon A Nachman

Masayuki Nagasawa

Joshua Nagler

Luca Nai Fovino

Timothy S Naimi

Samaneh Nakhaee

Vikram M Narayan

Keisuke Narita

Yvonne Nartey

Eleni Nastouli

Akina Natori

Jacob Nattermann

Anita Nelson

Ashley M Nelson

Shamim Nemati

Patrick Neven

Qin Xiang Ng

Anh Le Tuan Nguyen

Roch Nianogo

Claire Niedzwiedz

Louis Niessen

Hiromichi Niikawa

Naoki Niikura

Takahiro Niimura

Inger S Nijhof

Anton Nikouline

Per Nilsson

Abhishek Niroula

Janet E Njelesani

Kentaro Noda

Raywat Noiphithak

Claire Nollett

Gwendolyn S Norman

John Norrie

Robert Norton

Kin Israel Notarte

Seyed Mehdi Nouraie

Olivier Nunez

Francis Obare

Reginald Obiako

Chan-Young Ock

Alexander Odaibo

Camila Odio

Ekemini Ogbu

Graham David Ogle

Takeshi Okamoto

Andrew Olagunju

Verónica Olavarría

John Olajide Olawepo

Catherine Oldenburg

Timothée Olivier

Adam Olszewski

Kenji Omae

H A O'Mahen

David O'Neal

Daniela Oprea-Lager

Gokcen Orgul G

Alex Ortega-Loayza

Esteban Ortiz-Prado

Marie Österberg

Marlies Ostermann

Wim Otte

Martijn Oudijk

Carolyn Owen

Mayowa Owolabi

Gülin Öz

Enny Paixao

Johan Pallud

Daniel Pan

Xiong-Fei Pan

Huayang Pang

Xavier Paoletti

Aris T Papageorghiou

Manish Pareek

Eric Parent

Jean-Jacques Parienti

Carlos Luís Parra-Calderón

Sairam Parthasarathy

Timo Partonen

Patrizio Pasqualetti

Patrizio Pasqualetti

Lina R Patel

Dimitrios Patoulias

Angelo Maria Patti

Monica E Patton

Sarah Paul

Martin Paulus

Lillian Pazvakawambwa

Janet Peacock

Erin Pearson

Alisa Pedrana

Mirabel Pelton

Cheng-Yuan Peng

Zhiyong Peng

Melanie Penner

Richard Penson

Apostolos Perelas

A Perino

Carlo Federico Perno

Francesco Perrone

Thorsten Persigehl

Olumuyiwa James Peter

Jeff Zarp Petersen

Marek Petráš

Max Petrov

Elisa Pezzola

Mary E Phillips

Marco Picardi

Benjamin Alexander Pickwell-Smith

Sante Donato Pierdomenico

Filippo Pietrantonio

Valentina V Pilipenko

Jillian Pintye

Claudia Pisanu

Bertram Pitt

Dimitri Poddighe

Werner Poewe

Stanislas Pol

Marija M Polovina

Martí Pons-Òdena

Emanuele Pontali

Tatjana Potpara

Eero Poukka

Narendran Pradeep Kumar

Katrin H Preller

Robin Prescott

Jan Prins

Flavia Prodam

Christine Maria Prodinger

Destie Provenzano

Hans Prozesky

Fabio Puglisi

Julie Pulerwitz

Hua Qu

Eamonn Quigley

Gwendolyn Quinn

Camelia Radulescu

Luca Ragazzoni

Zahra Rahemi

Masoud Rahmati

Alessandra Raimondi

Diego Raimondo

Giovanni Raimondo

Roopali Rajput

Ranjith Ramasamy

Sara Ramella

Mika Ramet

Fahimeh Ramezani Tehrani

Pedro Ramirez

Elmars Rancans

Otavio Ranzani

Emanuel Raschi

Mohammad-Mahdi Rashidi

Luke Rasmussen

Francesco Raspagliesi

Kausik Ray

Gillian Ray-Barruel

Hassan Razvi

Gregory H Reaman

Travis Redd

Scott Regenbogen

Erin Reilly

Toralf Reimer

Daniela Renedo

Rungsun Rerknimitr

Ravi Retnakaran

Anna Reyners

Michael E Rezaee

Taeho Greg Rhee

Davide Giuseppe Ribaldone

Fabrizio Ricci

Henry Rice

Barbra Richardson

Paul Ridker

Stefan Rieken

Alan S Rigby

Lisa Riley

Lorenza Rimassa

Bobbie Rimel

Giancarlo Ripabelli

Helen Rizos

Alessandro Rizzo

Hilary Robbins

Bayard Roberts

Jessica Robinson-Papp

Marco Rocchi

Mónica Rodrigues

Paula Rodriguez-Miguelez

Leonardo Roever

Christian Rolfo

Manuel Romero-Gomez

Alexandra Rosa

Rafael Rosell

Sarah E Rosenbaum

Joshua Rosenblat

Annika Rosengren

Robert S Rosenson

Ali Rostami

Amihai Rottenstreich

James A Rowley

Campbell Roxburgh

Alicja Rudnicka

Piero Ruggenenti

Sebastian Rühling

Luis Ruilope

Piotr L Rutkowski

Frans Rutten

Mandy Ryan

Bushra Sabri

Emma Sacks

Lakkhana Sadaow

Sahar Saeedi Moghaddam

Marc Saez

Tomohiro Sakamoto

Zikria Saleem

Abraham Salinas-Miranda

Sam Salman

Kjell Åsmund Blix Salvesen

Arun Samidurai

Tarik Sammour

Miguel Ángel Sánchez Alemán

L Nelson Sanchez-Pinto

Robin Sanderson

Roberto Mario Santi

Damian Santomauro

Raul Santos

Amanda Veiga Sardeli

Md Samun Sarker

Reetta Satokari

Carol Saunders

Andrew Sayampanathan

Alyssa Sbarra

Giovanni Scambia

Bruno Scheller

Jeffrey F Scherrer

Claudia Schilling

Luregn Schlapbach

Sandra Schlegl

Mark Schleiss

Marcus Schmidt

Ricarda Schmidt

Wolf Schmidt

Thomas Schmitz

Antonius Schneider

Melissa J Schoelwer

Nancy Li Schoenborn

Reineke Schoot

Jeffrey Thomas Schouten

Anton Schreuder

Joachim Schüz

Brian Schwartz

Joseph Schwartz

Christopher Schwarz

Nora Schwotzer

Damiana Scuteri

Julian Segura

Rakesh Sehgal

Jakob Benedict Seidelin

Michael Seiden

David Seiffge

Thomas Sejersen

Mario Šekerija

Hen Yitzhak Sela

Geir Selbæk

Dave Selewski

Oliver Semler

Gaetano Settimo

Thomas Seufferlein

Esperança Sevene

Ahmat Sezer

Feng Sha

Muhammad Shaban

Aditi Shah

Gunjan Shah

Rory Shallis

Shahrokh Shariat

Mohit Sharma

Rajesh Sharma

Rajesh Sharma

Philip Shaw

Kiran Shekar

Aling Shen

Susan Shepherd

Ju-Fang Shi

Qingyang Shi

Si Shi

Yuxin Shi

Yusuke Shimakawa

Takaya Shimura

Yehuda Shoenfeld

Lawrence Shulman

Maximilian Siebert

Ilias Siempos

Márcio Roberto Silva

Valentina Silvestri

David Simmons

Otto Simonsson

Colin Simpson

Donald D Sin

Bhajan Singh

Harminder Singh

Navneet Singh

Rakesh Singh

Sarman Singh

Archana Singh-Manoux

Vassiliki Sinopoulou

Meghan Elizabeth Sise

Frederic Sitas

Sigrid Skanland

Rolv Skjaerven

Marry Smit

David Smith

Jaclyn Smith

James Smith

Kimberly R Smith

Robert Smith

Ryan Smith

Michael Smolensky

Joseph Smolich

Riccardo Soffietti

Mohammad Soleimani

María José Soler

Nico Sollmann

Jin Woo Song

Zhen Song

Guru Sonpavde

Vicente Soriano

Jacob Sosnoff

Valerie Speirs

Barbara Spellerberg

Anne Spinewine

Sarah Spinler

Anthony Spirito

Daniel Spratt

Nikola Sprigg

Derrick Ssewanyana

Joseph Standing

Natalie Staplin

Philip J Steer

Pasquale Stefanizzi

Barry Stein

Mark Stein

Arnulf Stenzl

Elizabeth R Stevens

Nathan Stevenson

William Stewart

Eric Stice

Charles A Stiller

Martin Stockler

Marie-Pierre St-Onge

Franc Strle

Walter Ludwig Strohmaier

Chien-Wei Su

Mohsan Subhani

S V Subramanian

Rebecca Jo Suckling

Mitsushige Sugimoto

Mitsushige Sugimoto

Olga A Sukocheva

Helen W Sullivan

Mary Sullivan

Hsin-Yun Sun

Liang Sun

Yan Sun

Ying-Shi Sun

Clara Suñer

Michael Swash

Eszter Szekely

George E Taffet

Scott Tagliaferri

Julien Taieb

Takeshi Takahashi

Toshimi Takano

Ryoji Takazawa

Emily Tam

Martin Tammemagi

Eng-King Tan

Peng Tan

Junko Tanaka

Yasuhito Tanaka

Dale Tang

Wei Tang

Weiming Tang

Wenxi Tang

Zhouping Tang

Kelan G Tantisira

Yong Tao

Maxime Taquet

Philip Tarr

Hirotaka Tashiro

Gregory E Tasian

Jennifer Taylor

Mark Taylor

Rod S Taylor

Helena Teede

Hélio Afonso Ghizoni Teive

Rohan M Telford

João Paulo Telles

Bethany R Tellor Pennington

Kevin ten Haaf

Ziwei Teng

Zhen Ling Teo

Martin Teraa

Larisa Tereshchenko

Amanda Termuhlen

Massimo Terzolo

Krishnansu Tewari

Ahmad Ahmadi Teymourlouei

Sophie Tezenas du Montcel

Varykina G Thackray

Gita Thanarajasingam

Tanayott Thaweethai

Gerhard Theron

Sathish Thirunavukkarasu

Dennis Thomas

Kate Nicole Thomas

Kim Thomas

Benjamin Thompson

Kelly Jane Thompson

Philip A Thompson

Benedikt Till

Jean-Francois Timsit

Angela A Tincani

Anna V Tinker

Corrado Tinterri

Satyendra K Tiwary

Geir Erland Tjönnfjord

Fumiharu Togo

Masakazu Toi

Adi Toledano-Shubi

Michelle Tollit

Bartłomiej Tomasik

Anli Tong

Zhihui Tong

L K Too

Vanessa Torbenson

Signe Torekov

Gianluca Tornese

Julie S Torode

Giovana Tardin Torrezan

Iakovos Toumazis

Marie Tournier

Francesco Tovoli

Hidenori Toyoda

Jay Traverse

Amery Treble-Barna

Suporn Treepongkaruna

Michelle Tregear

Dominic Trépel

Dmitry Tretiakow

Gianluca Trifiró

Vincenzo Trischitta

Maria Chiara Tronconi

Stephanie Truant

Ivan Trus

Sheng-Tzung Tsai

Elena Tsourdi

Amy Ong Tsui

Kunihiro Tsukasaki

Christopher Turnbull

Theogene Twagirumugabe

Gilad Twig

Stephen K Tyring

Athina Tzovara

Shahadat Uddin

Takamasa Ueno

Ugochinyere Vivian Ukah

Niels Uldbjerg

Chukwuma Umeokonkwo

Martin Underwood

Luka Ursic

Mai Utada

Olalekan Uthman

Louis J Vaickus

Michel Vaillant

B Vaira

Martina Valente

Laura Valenzuela

Alexander J C van Akkooi

Marleen Van Baak

Sebastiaan A S Van Der Bent

Marc van der Valk

Willemien van Driel

Marlies van Houten

F J van Kemenade

Hanneke Van Laarhoven

Ruth van Nispen

Max van Noesel

Annelies van Rie

Tjeerd P Van Staa

Ilse Vanhorebeek

Michael Varner

Anca Vasiliu

Prabhakaran Vasudevan

Lennert Veerman

Thirumalaisamy Velavan

Balasubramanian Venkatesh

Vidhya Venugopal

Johan Verhaeghe

Massimiliano Veroux

Adele Viguera

Liza Villaruz

Alessandro Villaschi

Renu K Virk

Leo Visser

Umberto Vitolo

Marc Vives

Arndt Vogel

Joshua Vogel

Elizabeth Volkmann

Mariza Vorster

Manon Vouga

Christiaan Vrints

Lars Wagner

Siegfried Wagner

Claire Wakefield

Michael Walker

Patricia Walker

Emma Wallace

Jack Wallace

Robert Wallis

Sebastian Walsh

Harriet Walter

Eugene Haydn Walters

Chen-Yu Wang

Faming Wang

He Wang

Hui Wang

Jaeyun Jane Wang

Jiantao Wang

Jihan Wang

Jingxuan Wang

Jinwei Wang

Kun Wang

Li San Wang

Liang Wang

Ling Wang

Qiming Wang

Shumin Wang

Wei Wang

Wei Wang

Xin Wang

Yihua Wang

Zhizhong Wang

Intan Mauli Warma Dewi

Saman Warnakulasuriya

Kei Watanabe

Grant Waterer

Brian Watermeyer

Michael A Weber

Marc Wehrli

Ke Wei

Zheng Wei

Anna Weiss

Paul Weiss

Joel S Weissman

Myrna Weissman

André Werneck

Bernhard Wernly

Heiman Wertheim

Colin West

Dirk Westermann

Amanda J Wheeler

Kate J White

Lori Wiener

Matthew Wiens

Annelies Wilder-Smith

Annelise Wilder-Smith

Malgorzata Wilinska

Robin Williams

Gregory Willis

Nina Wilson

Ingrid M Winship

Stuart S Winter

Bettina Winzeler

Andre Wirries

T Charles Witzel

Grace Wong

Stanley Sau Ching Wong

Hannah Wozniak

Che-Yuan Wu

Lin Wu

Qi-Jun Wu

Xian-Bo Wu

Xiang Wu

Xiaohua Wu

Yi-Long Wu

Yonghui Wu

Zhe Wu

Richard Wunderink

Immaculata Xess

Changfa Xia

Hongfei Xia

Gary Xiao

Xinhua Xiao

Yonghong Xiao

Feng Xie

Lin Xie

Wubin Xie

Yan Xie

Jin-Fu Xu

Wanghong Xu

Yanwen Xu

Yu Xu

Lalit Yadav

Clinton Yam

Toshinari Yamashita

Mika Yamauchi

Hajime Yamazaki

Hongmei Yan

Jinsong Yan

Haidi Yang

Ian Yang

Lejin Yang

Li Yang

Lin Yang

Min Yang

Minghui Yang

Pan-Chyr Yang

Ting Yang

Yanxiang Yang

Yuhan Yang

Xiaofei Ye

Chihjung Yeh

Chun-Nan Yeh

Nadir Yehya

Jonathan Yeung

Wenjun Yi

Musa Yilmaz

Minyue Yin

Guishuang Ying

Terry Yip

Vignan Yogendrakumar

Dong Keon Yon

Naohiro Yonemoto

Harry Yoon

Dokyoung You

Chiahung Yu

Honggang Yu

Jin-Tai Yu

Yongfu Yu

Chen Yuan

Ying Yuan

Yong Yuan

Courtney Yuen

Atil Yüksel

Rouhollah Zaboli

Ismail Zaed

Muhammad Sohail Zafar

Ashkan Zandi

Jean Pierre Zellweger

Hongmei Zeng

Hongmei Zeng

Na Zeng

Yongyi Zeng

Zhimin Min Zeng

Ababi Zergaw

Bo Zhang

Cheng Zhang

Feng Zhang

Fengqing Zhang

Haijun Zhang

Hua Zhang

Huabing Zhang

Jin Zhang

Luo Zhang

Ruijie Zhang

Teng Zhang

Xi Zhang

Xiaodong Zhang

Xing Zhang

Yaru Zhang

Zhongheng Zhang

Bin Zhao

Binghao Zhao

Chongke Zhao

Fang-Hui Zhao

Fengmin Zhao

Qianhua Zhao

Qun Zhao

Deqiang Zheng

Jiali Zheng

Weizeng Zheng

Lai-ping Zhong

Nanbert Zhong

Feng Zhou

Jianhua Zhou

Jiawei Zhou

Qing Zhou

Shi-Chong Zhou

Shouhao Zhou

Xiao Zhou

Zhen Zhou

Beibei Zhu

Guanghu Zhu

Jinzhou Zhu

Wengen Zhu

Xu Zhu

Zhengfei Zhu

Walter Zingg

Tommaso Zoerle

Mohammad Zoladl

Andres Zorrilla-Vaca

Zhiyong Zou

Zhuoru Zou

Gianvincenzo Zuccotti

